# Acute intracranial hemorrhage during the installation of the LICOX microdialysis system: A case report

**DOI:** 10.3892/mi.2024.163

**Published:** 2024-05-17

**Authors:** George Fotakopoulos, Ioannis Siasios, Charalampos Gatos, Vasiliki Epameinondas Georgakopoulou, Nikolaos Trakas, Pagona Sklapani, Konstantinos N. Fountas

**Affiliations:** 1 Department of Neurosurgery, General University Hospital of Larissa, 41221 Larissa, Greece; 2 Department of Neurosurgery, Papageorgiou Hospital, 56429 Thessaloniki, Greece; 3 Department of Pathophysiology, National and Kapodistrian University of Athens, 11527 Athens, Greece; 4 Department of Biochemistry, Sismanogleio Hospital, 15126 Athens, Greece

**Keywords:** LICOX system, neuromonitoring, intensive care unit, acute hemorrhage, complication

## Abstract

Neuro-monitoring is widely employed for the evaluation of intubated patients in the intensive care unit with stroke, severe head trauma, subarachnoid hemorrhage and/or hepatic encephalopathy. The present study reports the case of a patient with acute intracranial hemorrhage following the insertion of neuromonitoring catheters, which required surgical management. The patient was a 14-year-old male who sustained a severe traumatic brain injury and underwent a right-sided hemicraniectomy. During the installation of the neuromonitoring catheters, an acute hemorrhage was noted with a rapidly elevating intracranial pressure. A craniotomy was performed to identify and coagulate the injured cortical vessel. As demonstrated herein, the thorough evaluation of the clotting profile of the patient, a meticulous surgical technique and obtaining a post-insertion computed tomography scan may minimize the risk of any neuromonitoring-associated hemorrhagic complications.

## Introduction

Neuromonitoring constitutes an extensively employed diagnostic modality in intensive care units (ICUs) for the management of potentially fatal situations, such as closed head injury, intracerebral or subarachnoid hemorrhage, ischemic events and hydrocephalus. It is well known that all these pathological entities are related to secondary brain injury, which may lead to the development of edema and subsequent intracranial hypertension (1-9). Numerous methods of neuromonitoring are available and have been utilized in the clinical routine, ranging from intraparenchymal and intraventricular intracranial pressure (ICP) catheters to more advanced, multi-parametric monitoring systems that provide additional information regarding regional brain tissue oxygenation and temperature (6,9).

All the aforementioned neuromonitoring methods require surgical intervention for their insertion. However, it is widely accepted that they provide a more accurate evaluation of the neurological status of the patient compared with the existing non-invasive methods (9). Nevertheless, invasive neuromonitoring is associated with the risk of developing insertion-associated complications, such as infection, hemorrhage, cerebrospinal fluid leak, and the displacement or malfunction of the implanted device (9-12). Of note, the incidence of hemorrhage following the insertion of neuromonitoring systems has been reported to be relatively low. It is also notable that the majority of these hemorrhages are small, not clinically significant, and routinely do not require surgical evacuation (13-18).

The present study reports the case of a patient with intracranial hemorrhage following the insertion of an intraparenchymal neuromonitoring catheter, which required surgical intervention for its evacuation. The aim of the present study was to emphasize the importance of meticulous surgical maneuvers and the necessity of post-operative imaging studies in cases of neuromonitoring deterioration to avoid or mitigate such complications.

## Case report

**Figure 1:**
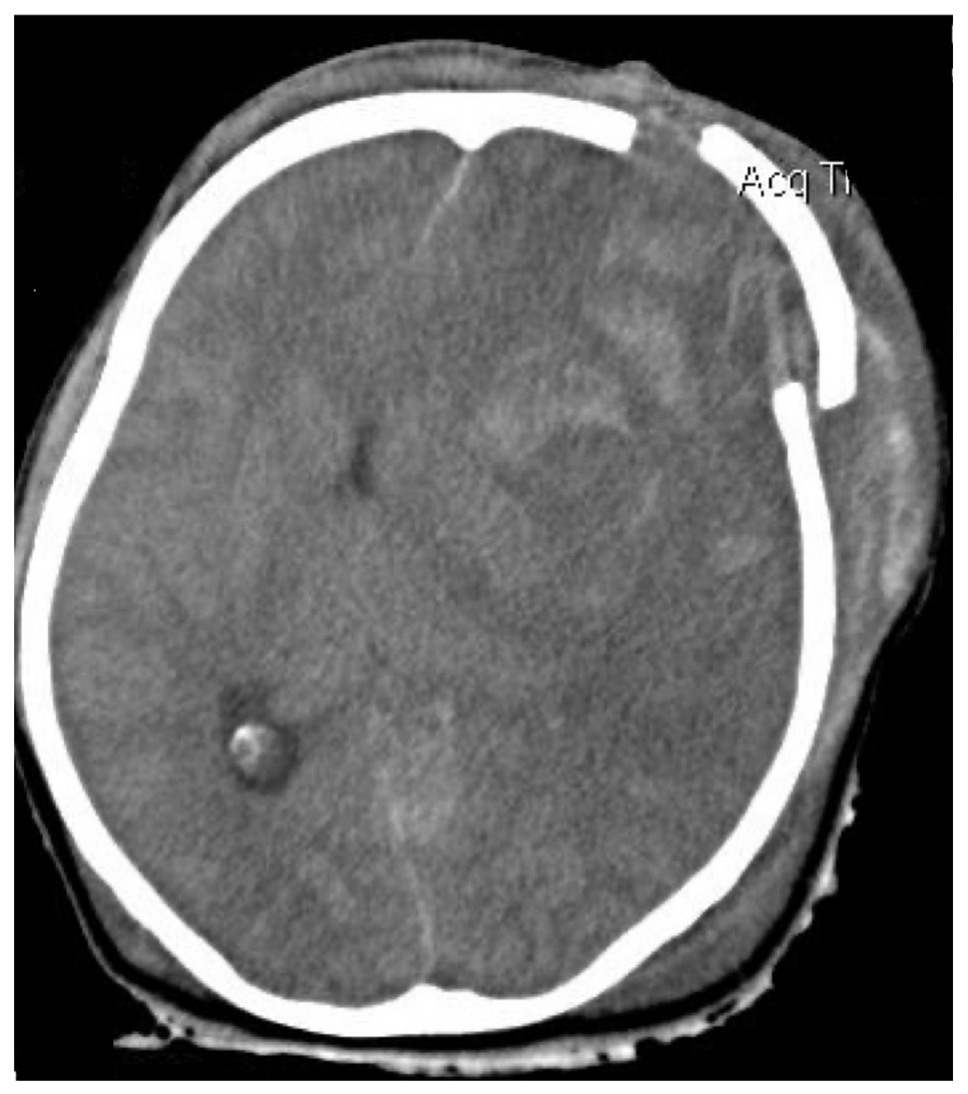
Axial non-contrast brain CT image illustrating a small right-sided fronto-temporal traumatic subdural hemorrhage, small bifrontal hemorrhagic cerebral contusions with the absence of midline shift, and multiple fractures of the frontal and right temporal bones of the skull without any marked deformation noted.

**Figure 2:**
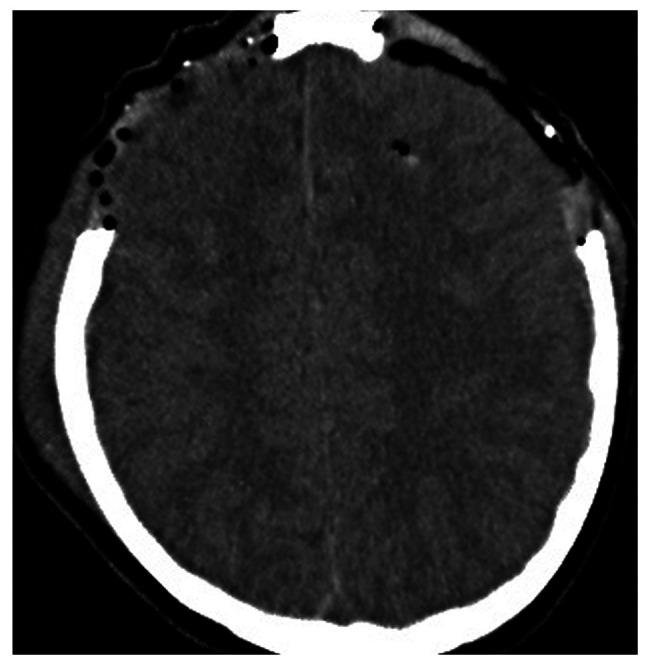
Axial non-contrast brain CT images illustrating the tips of the inserted neuromonitoring catheter following removal-the tip and the clot from the provoked hemorrhage are shown on the left side.

**Figure 3:**
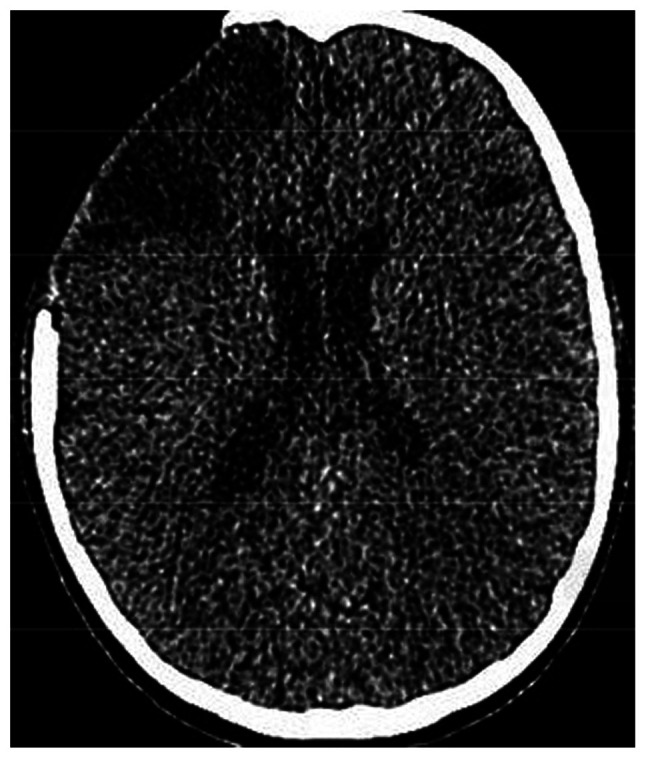
Axial non-contrast bone computed tomography image illustrating the right-sided hemicraniectomy, as well as an ischemic area in the right frontal lobe, after the performed procedure.

A 14-year-old male patient was transferred [he was intubated; intubation Glasgow Coma Scale (GCS), 9/15] to the General University Hospital of Larissa (Larissa, Greece) 2 h after he sustained a closed traumatic brain injury in a motorcycle traffic accident. He underwent an emergency computed tomography (CT) scan, which indicated a small right-sided fronto-temporal traumatic subdural hemorrhage, small bifrontal hemorrhagic cerebral contusions with the absence of midline shift, and multiple fractures of the frontal and right temporal bones of the skull without any marked deformation noted (Figs. 1 and 2). The patient had normal values of the routine clotting tests, such as international normalized ratio (INR, 1.12), prothrombin time (PT, 13.10 sec) and partial thromboplastin time (PTT, 34.30 sec) Initially, it was decided that an ICP monitoring system should be installed. Due to the rapid elevation of ICP (>25 mmHg) in the operating room, the patient underwent a right fronto-temporo-parietal decompressive craniectomy. It was decided that a LICOX monitoring system should be installed, according to the head trauma ICU protocol. Following bolt insertion and during catheter installation, high-pressure bloody fluid was coming out of the three lumens. The surgeons removed the complex of the lumens and washed the bolt without noticing any reduction in blood production. They repeated this procedure for 10 min in order to cease the bleeding; however, the hemorrhage persisted, leading to an increase in ICP (>20 mmHg). Therefore, the surgical team removed the bolt and removed the surrounding segment of the right frontal bone with the drill. After recognizing the bleeding vessel located on the right frontal cortical area, they cauterized it with the use of a bipolar cautery. Furthermore, the surgeons removed a formatted acute epidural-subdural hematoma on the site of the hemi-craniectomy. The patient was then transferred to the ICU. Upon his awakening 48 h after the procedure, his GCS was 15/15, and he was neurologically intact (Fig. 3). He had an uneventful post-operative course, and later on, he underwent cranioplasty surgery. The patient had a 2-year follow-up and he was neurologically intact.

## Discussion

There are several catheters that can be used for several aspects of neuromonitoring, such as subdural, epidural, intraparenchymal and intraventricular ICP catheters, brain tissue oxygen catheters, temperature catheters, micro-dialysis catheters, and cerebral blood flow catheters, which allow an accurate evaluation of sedated patients in critical neurological conditions (9,19). The employment of multi-parametric neuromonitoring requires the insertion of multiple-lumen bolts to avoid separate insertion sites, minimizing the risk of any insertion-associated complications (20). Of note, two different techniques of catheter insertion exist: The open technique, in which the insertion is performed under direct vision, and the twist-drill insertion technique, which allows for the insertion to be made through a small opening. Each one of these techniques has advantages and disadvantages (20). The major advantage of the open approach is the selection of an avascular cortical area as an entry point. Furthermore, the open approach allows for the control of any cortical vessel bleeding during the insertion. The major drawback is that it requires a larger skin incision, a small-size craniotomy, factors that may increase the risk of infection, and, of course, an increased operative time. On the other hand, a twist-drill approach minimizes the surgical trauma, the risk of infection and the procedural time; however, on rare occasions (as in the case presented herein), a marked cortical hemorrhage, which cannot be controlled, may be encountered (20). A useful technical tip, applicable to both approaches, is the slow twisting of the catheter during its insertion, so that any adjacent tiny vessels will be pushed away and will not be injured (20).

It is well-described in the pertinent literature that two types of hemorrhage can be provoked by catheter insertion: A cortical or an intraparenchymal, due to the mechanical disruption of a cortical or a subcortical vessel, respectively. Cortical bleeding can be managed in the vast majority of cases with warm saline irrigation, or on rare occasions, the identification and coagulation of the injured vessel are required (21,22). Intraparenchymal bleeding has a different pattern of development. It usually expands more slowly than cortical hemorrhages, and it usually requires no intervention unless it compresses the adjacent brain parenchyma and causes an increase in the ICP. In the case described in the present study, the hemorrhage was intraparenchymal and was detected immediately following surgery on the obtained CT scan. The presence of intraventricular hemorrhage along with the occurrence of a cerebrovascular ischemic event at such a young age raises the suspicion of clinically silent thrombophilia, despite the normal values of the routine clotting tests, such as international normalized ratio (INR), prothrombin time (PT), partial thromboplastin time (PTT) of the patient. Indeed, the patient was further evaluated during his hospitalization and was found to be a homozygote for the A1298C point mutation of the MTHFR gene and a heterozygote for the 4G/5G polymorphism of the PAI-1 gene (21,22). These findings may well explain the occurrence of an ischemic stroke at a young age, as well as the occurrence of a large hemorrhage after the monitoring of the catheter insertion (21,22). In addition, in the case presented herein, the hemorrhage was due to a cortical vessel trauma, which was found and coagulated with no further consequences for the patient.

The incidence of hemorrhagic complications, such as epidural and/or subdural hematomas, ventricular hemorrhage, or intraparenchymal hemorrhagic contusions following the insertion of invasive neuromonitoring catheters, is generally low and varies with the type of the monitoring catheter. More precisely, over the past decade, the reported rates of hemorrhagic complications following the placement of intraparenchymal ICP catheters have been shown to range from 0.6 to 15.6% (13,23,24). The incidence of hemorrhagic events during the placement of an external ventriculostomy ranges from 0.7 to 46.2% (18,25-27). The incidence of hemorrhagic contusions associated with the insertion of a multi-lumen monitoring device has been reported to be <2% (20,28). Rarely, these hemorrhages require surgical intervention and evacuation, while in the vast majority of cases, observation and conservative management are sufficient (26,27-29). On the other hand, the risk of developing intracranial hemorrhage associated with neuromonitoring catheter insertion has been reported to be 0.2-4.4% and the risk of insertion-associated complications, such as infection cerebrospinal fluid leak, and the displacement or malfunction of the implanted device has been shown to be very low (9-12). Thus, the use of neuromonitoring catheter insertion is beneficial to avoid or mitigate such complications.

Previous studies have demonstrated that the placement of ICP monitoring catheters may be successfully performed by non-neurosurgeons, such as trauma surgeons or intensive care physicians (14,30,31). The documented rates of procedure-related complications are comparable to those of the neurosurgical series. As previously mentioned, hemorrhagic complications following the insertion of a monitoring device are of minor clinical significance in the vast majority of cases. However, in some extremely rare occasions, further surgical intervention may be required for the management of an expanding hematoma, as in the presented cases. In addition, a more comprehensive discussion of risk factors and preventive strategies is warranted, except for mentioning the potential role of thorough clotting profile evaluation, meticulous surgical technique, and post-insertion CT scans in minimizing hemorrhagic complications. This may include pre-existing coagulopathy or thrombophilia; anticoagulant or antiplatelet therapy; intraoperative blood pressure control and anesthetic management; correct selection of the catheter size, material, and insertion depth; the use of image guidance (e.g., neuronavigation) during catheter placement; protocols for immediate post-insertion neurological assessment and imaging. In such cases, the presence of a skilled neurosurgeon is of paramount clinical, but also of medico-legal importance for the proper management of any complications in a timely manner.

In conclusion, invasive neuromonitoring is useful for the management of critical patients with intracranial pathology in the neuro-ICUs. Although the risk of developing a rapidly evolving acute hemorrhage is relatively low, the occurrence of such an event may be devastating for the patient. In these rare cases, the presence of a neurosurgeon is of paramount importance. The importance of a thorough evaluation of the clotting profile of these patients, the employment of careful surgical maneuvers, and the obtaining of a post-insertion imaging CT scan are apparent for avoiding or mitigating any hemorrhagic complications. In addition, the case described in the present study may suggest the development of standardized protocols for post-insertion monitoring and imaging to facilitate early detection and management of intracranial hemorrhage.

## Data Availability

The datasets used and/or analyzed during the current study are available from the corresponding author on reasonable request.
